# Tapping Ovarian Tumors

**Published:** 1876-05

**Authors:** M. B. Wright

**Affiliations:** Late Professor of Obstetrics in Ohio Medical College; Cincinnati


### TAPPING OVARIAN TUMORS.

By M. B. WRIGHT, M.D.,

Late Professor of Obstetrics in Ohio Medical College.

I have derived pleasure and profit from a perusal of Dr. Byford's paper on ovarian cysts. It gives a comprehensive view of the entire subject, and presents very clearly individual sentiments, respecting the utility of operations and their kind. What shall be done with the more solid tumors is a question admitting direct replies. Remove them bodily, or let them alone. The treatment of these tumors by medicines, such as act on the kidneys, intestines and skin, or by counter-irritants, is of doubtful efficacy. My own belief is, that their long continuance impairs the general system, and tends to increase rather than retard the growth of the tumor. A well devised tonic treatment, with he introduction of a cotton ball saturated with tinct. iodine, and the fluid ext. ergot, and glycerine, one part of the former to two parts each of the latter, have given much satisfaction before the tumors had attained any considerable size. But my object at this time is rather to present a case of single cyst, cured seemingly by tapping alone.

S. P., aged 17, single, entered the Cincinnati Hospital Aug. 12, 1874. Family history good ; parents, substantial Germans.

Two years ago, she observed a degree of tumefaction in the lower and pubic portion of the abdomen. For a time it was attributed to flatulency, and for this treated. The abdomen increased gradually in size, and without much inconvenience until May, 1874, when, from oppressed breathing, it was deemed best to resort to tapping. A large quantity of fluid was drawn off (quantity not given) to the great relief of the patient. No pain was experienced during the accumulation of fluid. Now and then, however, subsequent to tapping, lancinating pains were felt in the abdomen, and the catamenial function was suspended during two periods.

When the patient entered the hospital, three months after the tapping, the enlarged abdomen measured thirty-five inches. She also complained of pain in the left ovarian region.

August 31. Breathing is somewhat oppressed ; measurement, 38$\frac{3}{4}$ inches ; fluctuation is distinct ; there is dullness on percussion, except in the lumbar regions, where there is distinct resonance. The swelling has a little more prominence in the left than right iliac region. There is an unusual firmness in the left cul de sac of the vagina.

The diagnosis is a unilocular ovarian cyst.

The patient had been advised to enter the hospital, and submit to an operation. The summer heat was unfavorable for an operation as hazardous as that of ovariotomy. Tapping presented three points for favorable consideration : the comfort of the patient, a more accurate diagnosis as to the solid elements of the swelling, and the most judicious treatment. In view of the general condition of the patient, as well as the diagnosis, I felt strongly urged to test the efficacy of tapping, as a prominent means of cure. Accordingly, I drew from the cyst, by means of a bistoury and gum-elastic tube, inserted in the linea alba, twenty-six pints of a dense ropy fluid. Before the complete emptying of the cyst, the patient became slightly faint, from which she soon rallied by slight stimulation. A compress and bandage were applied, and the patient ordered to bed. In two or three days she was walking about, and on October 26 she left the hospital.

She was re-admitted April 5, 1875, with a prominent abdomen, although not as large as at the last tapping. Her bowels were inactive, and the kidneys were not secreting well. Her appetite was impaired, and her strength had failed. Under the influence of purgatives, diuretics and tonics, a better state of health was established.

May 28, she was again tapped, with sensible relief.

June 28, she left the hospital, since which time she has increased in flesh and strength, and up to this time, Feb. 10, 1876, there has been no perceptible accumulation of fluid.

This case afforded just ground for discussion. On the one hand it was claimed, that, ultimately, a more radical operation would be demanded, and that while the patient remained in general good health, success might be reasonably anticipated. On the other hand there was before me a young woman of fine physical development, and who was suffering in no way except from her bulk. There was no evidence of a solid tumor. She could be relieved by a simple operation, and this could be performed from time to time without danger. The re-accumulation of fluid was at long intervals. The hope of final success seemed justified. And the question, how long will the patient live? was not pertinent nor pressing. Again, I am in favor of a partial or complete evacuation of the cyst before an attempt is made for its removal by incision.

The following case presents some points of interest as taken from the hospital record:

E. L., widow, aged 42. Has been pregnant seven times, the last six pregnancies terminating prematurely. Attributes her miscarriages to hard work—washing, ironing, etc. Has not been pregnant for fifteen years.

About four years ago, she observed a swelling near and below the umbilicus, which has been gradually increasing. There is difficult breathing in any position, but she cannot sleep in the recumbent posture. The abdomen is of immense size and irregular upon the surface. The diagnosis is, a more or less solid tumor, with many cysts of varying dimensions. Fluctuation, here and there, was observed in the larger cysts near the surface. One was punctured below the umbilicus, and two between the umbilicus and superior spinous process of the ilium, on the right side. The aggregate amount of fluid drawn, measured two hundred ounces. The fluid in the three sacs differed both in color and consistence.

The next day, June 21, the report was that she had slept soundly in any position, and that she was comparatively at ease.

June 23. The patient is walking about the ward, cheerful and free from pain.

About the first of August she was regularly transferred to the care of my associate and successor, Dr. Tate. Notwithstanding the patient suffered the inconvenience of bulk only, she expressed a strong desire for the removal of the tumor. Accordingly, on a cool day in November, Dr. Tate performed ovariotomy. Extensive and strong adhesions were found, complicating the case, and rendering it almost necessarily fatal.

The operation, deliberately and skillfully performed, was followed by death on the third day. The tumor weighed about twenty-five pounds.

Every operation for the removal of ovarian tumors, whatever may be their character, is attended with hazard. I have witnessed an expert extraction of a single cyst, without adhesions, with a fair state of general health, and with favorable surroundings, which terminated fatally, contrary to all reasonable expectation.

Again, the operator has taken up his knife with fear and trembling, feeling that death was in every incision, and yet the result has been favorable. Hence, the diagnosis of a case and the effects of an operation are vastly different.

Those who are opposed to tapping, carry their prejudices so far as to declare that it renders a radical operation uncertain, if not positively fatal. There has been nothing within my limited experience that justifies any such assumption. I have tapped and seen tapped many cases of single and multiple cysts, with decided benefit, and without injury in a single case. Does inflammation result from tapping, and thus add gravity to the disease and the operation? It is scarcely possible. The punctures, for illustration, in the case above, had nothing to do with the inflammation and adhesions already described. They were free from even a blush of inflammation. It is urged that the sacs refill, and often rapidly. Suppose they do, they are no more unpromising than the original cysts, and if need be, tap again.

Those who have operated most frequently, and are most distinguished for success, not only desire quietude, pure air, and a "preparation" favorable to the healthy performance of the functions, but a bracing temperature as auxiliary to success. Summer heat is prejudicial to all grave operations. Now, suppose a tumor, partly if not wholly cystic, should create discomfort almost beyond endurance, and the season should be unfavorable for an operation, would it not be better to lessen the volume of the tumor by tapping, than to attempt a radical cure? Palliation, if possible, is demanded in every case, curable or incurable.

CINCINNATI, Jan. 25, 1876.

